# Evaluation of the efficacy and safety of oral *N*‐acetylcysteine in patients with COVID‐19 receiving the routine antiviral and hydroxychloroquine protocol: A randomized controlled clinical trial

**DOI:** 10.1002/iid3.1083

**Published:** 2023-11-20

**Authors:** Najmolsadat Atefi, Azadeh Goodarzi, Taghi Riahi, Niloofar Khodabandehloo, Mahshid Talebi Taher, Niloufar Najar Nobari, Farnoosh Seirafianpour, Zeinab Mahdi, Amir Baghestani, Rohollah Valizadeh

**Affiliations:** ^1^ Department of Dermatology, Rasool Akram Medical Complex Clinical Research Development Center (RCRDC), School of Medicine Iran University of Medical Sciences Tehran Iran; ^2^ Department of Internal Medicine, School of Medicine Iran University of Medical Sciences Tehran Iran; ^3^ Department of Geriatric Medicine, School of Medicine Iran University of Medical Sciences Tehran Iran; ^4^ Department of Infectious Disease, School of Medicine, Antimicrobial Resistance Research Center, Immunology and Infectious Disease Research Institute Iran University of Medical Sciences Tehran Iran; ^5^ Razi Drug Research Center Iran University of Medical Sciences Tehran Iran; ^6^ Department of General Medicine, Rasool Akram Medical Complex, School of Medicine Iran University of Medical Sciences Tehran Iran; ^7^ Urmia University of Medical Sciences Urmia Iran

**Keywords:** antiviral, atazanavir, COVID‐19, hydroxychloroquine, Kaletra, lopinavir, NAC, *N*‐acetylcysteine, ritonavir

## Abstract

**Background:**

The current absence of gold‐standard or all‐aspect favorable therapies for COVID‐19 renders a focus on multipotential drugs proposed to prevent or treat this infection or ameliorate its signs and symptoms vitally important. The present well‐designed randomized controlled trial (RCT) sought to evaluate the efficacy and safety of N‐acetylcysteine (NAC) as adjuvant therapy for 60 hospitalized Iranian patients with COVID‐19.

**Methods:**

Two 30‐person diets, comprising 15 single diets of Kaletra (lopinavir/ritonavir) + hydroxychloroquine (HCQ) with/without NAC (600 mg TDS) and atazanavir/ritonavir + HCQ with/without NAC (600 mg TDS), were administered in the study.

**Results:**

At the end of the study, a further decrease in C‐reactive protein was observed in the NAC group (*P* = 0.008), and no death occurred in the atazanavir/ritonavir + HCQ + NAC group, showing that the combination of these drugs may reduce mortality. The atazanavir/ritonavir + HCQ and atazanavir/ritonavir + NAC groups exhibited the highest O_2_ saturation at the end of the study and a significant rise in O_2_ saturation following intervention commencement, including NAC (*P* > 0.05). Accordingly, oral or intravenous NAC, if indicated, may enhance O_2_ saturation, blunt the inflammation trend (by reducing C‐reactive protein), and lower mortality in hospitalized patients with COVID‐19.

**Conclusion:**

The NAC could be more effective as prophylactic or adjuvant therapy in stable non‐severe cases of COVID‐19 with a particularly positive role in the augmentation of O_2_ saturation and faster reduction of the CRP level and inflammation or could be effective for better controlling of COVID‐19 or its therapy‐related side effects.

## Graphical abstract

The present well‐designed randomized controlled trial sought to evaluate the efficacy and safety of *N*‐acetylcysteine (NAC) as adjuvant therapy in hospitalized Iranian patients with coronavirus disease 2019 (COVID‐19). The NAC could be more effective as prophylactic or adjuvant therapy in stable and nonsevere cases of COVID‐19 with a particularly positive role in the augmentation of O_2_ saturation and faster reduction of the C‐reactive protein level and inflammation.

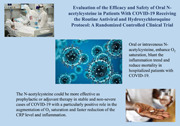

## 1 INTRODUCTION

On March 11, the World Health Organization (WHO) announced the outbreak of coronavirus disease 2019 (COVID‐19) and declared it to be an epidemic.^1^, ^2^ The virus is transmitted through respiratory droplets or aerosols.^3^ One of the theories concerning the coronavirus pathogenesis is that the virus binds to host cells through angiotensin‐converting enzyme 2 (ACE2). ACE2 is expressed by the epithelial cells of the lung, intestine, kidney, and blood vessels.^4^ Diabetes, ACE inhibitors, and angiotensin II receptor blockers, which are used for hypertension control, increase ACE2 expression and COVID‐19 risk.^4^ The symptoms of COVID‐19 include dry coughs; malaise; fever; dyspnea; multiorgan failure; acute respiratory distress syndrome (ARDS) requiring mechanical ventilation and oxygen therapy in the intensive care unit (ICU); coagulopathy with thrombosis; systemic manifestations such as sepsis, septic shocks, and multiorgan dysfunction syndrome; and mucocutaneous involvement.^5^, ^6^, ^7^, ^8^, ^9^ Inflammatory responses, cytokine storms, and chemokines are critical issues allied to the complications of COVID‐19.^10^, ^11^ About 33% of the patients with COVID‐19 require ICU admission, with a mortality rate of 20% reported in some investigations.^12^, ^13^ Additionally, a mortality rate of 49.0% has been reported among critical patients with comorbid cardiovascular diseases, hypertension, diabetes, chronic respiratory diseases, or cancer.^12^ Polymerase chain reaction (PCR) tests of the upper respiratory tract samples, lung computed tomography (CT) scans, and blood tests are accepted by the WHO for the diagnosis of COVID‐19.^3^, ^14^ *N*‐acetylcysteine (NAC) is a multipotential drug suggested by the literature for the prevention and treatment of COVID‐19.^1^, ^15^, ^16^, ^17^, ^18^, ^19^, ^20^, ^21^, ^22^, ^23^, ^24^ NAC is an antioxidant with a wide variety of use in different medical conditions such as pulmonary disorders, cystic fibrosis, bronchitis, chronic obstructive pulmonary disease, and pneumonia.^6^, ^24^ Evidence indicates the important roles of NAC in the prevention and treatment of COVID‐19.^24^, ^25^, ^26^ COVID‐19 can manifest itself through neurological disorders such as Guillain–Barre syndrome, seizure, headache, and stroke.^27^ NAC is capable of exerting protective effects on the nervous system and helps prevent or treat these manifestations.^5^ Liver failure can develop in patients with COVID‐19 for several reasons, including metabolic acidosis and complications induced by certain drugs such as remdesivir, which is one of the most commonly used drugs in these patients. In this regard, one of the most well‐known effects of NAC is the prevention and treatment of hepatotoxicity.^28^, ^29^, ^30^

### 1.1 A gap of knowledge

In spite of the multitude of research dedicated to COVID‐19, a definitive and universally accepted treatment for this ailment remains elusive. As a result, current strategies primarily revolve around providing supportive care. The most effective approach to tackling the illness continues to be prevention, with global vaccination efforts already in progress. However, it is important to note that instances of COVID‐19‐linked infections and fatalities persistently persist. Most COVID‐19 supportive drugs modulate the immune system to regulate inflammatory storms.^5^ Many of the immune modulators have immunosuppressive properties that may not work properly in viral disorders.^5^ NAC is one of the few immune modulators without immunosuppressive properties.^31^ However, all the articles suggesting the use of NAC in the treatment of COVID‐19 recommend further well‐designed randomized controlled clinical trials (RCTs).

### 1.2 Aim

This RCT, conducted under double‐blind conditions (with both secondary assessors and analysts unaware of the details), aimed to assess the impact of oral NAC in the management of COVID‐19 patients admitted to the hospital.

While there's evidence suggesting notable advantages of NAC for individuals with mild COVID‐19 before hospitalization, this specific investigation concentrated exclusively on patients already in the hospital setting. This study stands out as one of the meticulously planned RCTs aimed at gauging the effectiveness and safety of NAC as supplementary treatment for hospitalized COVID‐19 patients.

## 2 METHODS

### 2.1 Design and settings

The present double‐blind RCT was performed in Rasool Akram Medical Complex, Tehran, Iran, on 60 patients with COVID‐19. The diagnosis of COVID‐19 was established according to the opinion of the treating physician, based on clinical signs and PCR or paraclinical or laboratory findings.

### 2.2 Sampling and allocation

The sampling convenience method was employed. Eligible participants were classified by stratified blocked randomization and based on diet therapy (four regimens). Thereafter, they were randomly assigned to either the intervention group or the routine treatment regimen group. Randomization was done separately within each group. The size of the blocks was four; in other words, two allocations to the intervention group and two allocations to the routine treatment group were considered.

### 2.3 Eligibility criteria

The indication for hospitalization according to the national protocol was as follows: fever above 39° or being toxic in the examination, respiratory distress, the use of respiratory muscle relaxants, the use of suprasternal or intercostal retraction, a respiratory rate greater than 30/min, a heart rate higher than 120 beats/min, a peripheral blood O_2_ saturation level less than 93%, having an underlying disease (e.g., diabetes, hypertension, heart failure, immune system disorders, renal or hepatic impairment, a history of asthma or chronic obstructive pulmonary disease, and smoking), age over 50 years in the case of being symptomatic, and the involvement of one‐third of 3–5 pulmonary lobes. The criteria for exclusion encompassed individuals who were minors, those with unstable vital signs, individuals either already intubated or requiring intubation, those with diminished levels of consciousness, a respiratory rate exceeding 24, blood pressure readings below 90/60 mm Hg, showing multilobular infiltration in CT scans or chest X‐rays, experiencing persistent hypoxia, pregnant or nursing individuals, and those with past hypersensitivity reactions to NAC or medications containing glutathione. The withdrawal criteria were comprised of drug intolerance, severe complications probably related to NAC during treatment, and unwillingness to continue collaboration with the study at any point and for any reason.

### 2.4 Interventions and follow‐up

Two 30‐person diets, comprising 15 single diets of Kaletra (lopinavir/ritonavir) (LOPINAVIR/RITONAVIR Aurobindo 200/50 MG Tablet) + hydroxychloroquine (HCQ) (HYDROXYCHLOROQUINE AMIN 200MG TAB) with/without NAC (ACETYLCYSTEINE‐HAKIM 600 MG EFF TAB) (600 mg total dissolved solids [TDS]) and atazanavir/ritonavir (atazanavir (300 mg) + ritonavir (100 mg), India) + HCQ with/without NAC (600 mg TDS), were administered in the study. Sixty patients completed the study (15 patient: Kaletra + HCQ/15 patient: Kaletra + HCQ + NAC/15 patient: atazanavir/ritonavir + HCQ/15 patient: atazanavir/ritonavir + HCQ + NAC). The control and intervention groups received the national protocol treatment, and NAC was added to the treatment of the intervention group. Since the eligible patients had no contraindications for NAC, the protocol was 600 mg orally every 8 h for 14 days.

### 2.5 Blinding

The secondary assessor and the data analyst were blinded to the treatment regimens.

### 2.6 Paraclinical data

Laboratory parameters were evaluated by using the peripheral blood samples of the patients. Additionally, lactate dehydrogenase (LDH), tumor necrosis factor‐α (TNF‐α), interleukin‐6 (IL‐6), complete blood count (CBC), erythrocyte sedimentation rate (ESR), and C‐reactive protein (CRP) tests were performed daily for the patients, and the course of laboratory changes was monitored. Radiological examinations of the lungs were performed by CT scanning twice: at admission and discharge, and differences in radiological findings were recorded and compared. In the patients with minor underlying problems or gastrointestinal intolerance, 600 mg of oral tablets every 12 or 24 h were given.

### 2.7 Response to treatment criteria

- Time of improvement in symptoms such as coughs, shortness of breath, and lethargy.
- Improvements in O_2_ saturation without changes in the treatment protocol and reductions in the need for O_2_.
- Duration of hospitalization according to the course of symptom improvement.
- Readmission after discharge.
- Serial evaluations of laboratory parameters, consisting of LDH, TNF‐α, IL‐6, CBC, ESR, and CRP, and comparison of parameters at hospitalization, during hospitalization, and at discharge.
- Investigation of changes in anti‐inflammatory parameters.
- Examination of radiological changes at the beginning of hospitalization and during hospitalization.
- Need for ICU admission during hospitalization.

### 2.8 Primary and secondary outcomes

The primary outcomes of the study were the efficacy and side effects of NAC, and the secondary outcomes were drug tolerance and treatment satisfaction. The effectiveness of treatment was evaluated according to the duration of hospitalization; improvement in O_2_ saturation, laboratory and paraclinical findings, and clinical symptoms; and the assessment of complications based on a questionnaire.

### 2.9 Ethical considerations

The research adhered to the tenets of the Declaration of Helsinki. The study protocol was approved by the Ethics Committee of Iran University of Medical Sciences (ethical code #IR.IUMS.REC.1399.206 registered on August 16, 2020), and the study protocol was registered in the Iranian Registry of Clinical Trials (#IRCT20200623047897N1; https://en.irct.ir/trial/49277). Written informed consent was obtained from all the patients.

### 2.10 Statistical analysis

After entering the required data from the patient's records, the data were analyzed using SPSS version 21. Besides, descriptive data for continuous variables and qualitative statistics were used as bar charts and tables. One‐way Analysis of variance (ANOVA) repeated measure was used to compare the quantitative variables among groups as well as linear regression was applied to predict the factors affecting the length of stay at the ICU. *p* Value less than .05 was considered significant.

## 3 RESULTS

In this research study, the average age of the patients was 57.82 years with a standard deviation of 18.23 years, and the average duration of hospitalization was 10.13 days with a standard deviation of 6.07 days. In terms of gender distribution, 31 patients (51.7%) were female, and there were no statistically significant differences observed between the various groups. Detailed clinical and paraclinical characteristics of the study population at both admission and discharge are presented in Tables 1 and 2. Notably, the analysis revealed differences among the four groups in terms of certain parameters at discharge.

**Table 1:** Clinical and paraclinical characteristics of the study population admission.

Variable	Mean	SD	*F* a	*p*‐Value^b^
Hospitalization days				
Kaletra + HCQ	10.60	7.298	2.347	.082
Kaletra + HCQ + NAC	11.87	4.941		
Atazanavir/ritonavir + HCQ	6.73	3.058		
Atazanavir/ritonavir + HCQ + NAC	11.33	7.158		
ICU days				
Kaletra + HCQ	3.47	6.174	1.714	.174
Kaletra + HCQ + NAC	2.47	4.704		
Atazanavir/ritonavir + HCQ	0.00	0.000		
Atazanavir/ritonavir + HCQ + NAC	1.80	3.783		
WBC				
Kaletra + HCQ	8.5000	5.26661	0.963	.417
Kaletra + HCQ + NAC	10.0200	6.00990		
Atazanavir/ritonavir + HCQ	12.4533	17.83836		
Atazanavir/ritonavir + HCQ + NAC	6.4667	3.88471		
diff_segment				
Kaletra + HCQ	68.1467	28.47184	1.210	.316
Kaletra + HCQ + NAC	80.7462	8.23120		
Atazanavir/ritonavir + HCQ	74.7231	9.10386		
Atazanavir/ritonavir + HCQ + NAC	73.5714	13.89363		
diff_lymphocyte				
Kaletra + HCQ	16.3000	9.91396	1.679	.184
Kaletra + HCQ + NAC	15.5231	8.01105		
Atazanavir/ritonavir + HCQ	22.1154	9.19464		
Atazanavir/ritonavir + HCQ + NAC	21.2071	10.23782		
seg_lymph_ratio				
Kaletra + HCQ	7.8954	6.13541	3.007	.039
Kaletra + HCQ + NAC	6.6869	3.82775		
Atazanavir/ritonavir + HCQ	4.0519	1.88692		
Atazanavir/ritonavir + HCQ + NAC	4.3714	2.22906		
RBC				
Kaletra + HCQ	4.5567	0.62525	0.958	.419
Kaletra + HCQ + NAC	4.7333	0.59904		
Atazanavir/ritonavir + HCQ	4.4107	0.94044		
Atazanavir/ritonavir + HCQ + NAC	4.3373	0.53022		
HGB				
Kaletra + HCQ	12.9533	1.61946	0.212	.888
Kaletra + HCQ + NAC	13.3475	1.80408		
Atazanavir/ritonavir + HCQ	12.9467	2.96211		
Atazanavir/ritonavir + HCQ + NAC	12.7800	1.32891		
PLT				
Kaletra + HCQ	184.9333	81.10528	0.469	.705
Kaletra + HCQ + NAC	209.1333	135.86436		
Atazanavir/ritonavir + HCQ	169.2000	84.42934		
Atazanavir/ritonavir + HCQ + NAC	202.6000	97.13378		
ESR				
Kaletra + HCQ	50.7273	23.57580	1.492	.233
Kaletra + HCQ + NAC	58.5000	25.39685		
Atazanavir/ritonavir + HCQ	36.2222	23.28507		
Atazanavir/ritonavir + HCQ + NAC	55.4444	28.08964		
CRP				
Kaletra + HCQ	38.6711	14.42544	4.434	.009
Kaletra + HCQ + NAC	48.0080	0.00422		
Atazanavir/ritonavir + HCQ	26.4983	19.56495		
Atazanavir/ritonavir + HCQ + NAC	28.8020	17.16058		
PT				
Kaletra + HCQ	14.9071	2.37567	0.633	.597
Kaletra + HCQ + NAC	14.4000	1.78282		
Atazanavir/ritonavir + HCQ	14.1500	1.15719		
Atazanavir/ritonavir + HCQ + NAC	14.0867	1.38248		
INR				
Kaletra + HCQ	1.1936	0.27712	0.259	.855
Kaletra + HCQ + NAC	1.1443	0.21429		
Atazanavir/ritonavir + HCQ	1.1500	0.14460		
Atazanavir/ritonavir + HCQ + NAC	1.1267	0.17915		
PTT				
Kaletra + HCQ	34.2143	4.02260	3.853	.015
Kaletra + HCQ + NAC	40.0714	9.16065		
Atazanavir/ritonavir + HCQ	33.5500	4.31414		
Atazanavir/ritonavir + HCQ + NAC	33.6600	4.70741		
BUN				
Kaletra + HCQ	26.6000	24.81589	2.045	.118
Kaletra + HCQ + NAC	17.1429	12.30295		
Atazanavir/ritonavir + HCQ	17.2000	9.90815		
Atazanavir/ritonavir + HCQ + NAC	13.4667	7.15009		
Cr				
Kaletra + HCQ	1.5400	1.05343	1.932	.135
Kaletra + HCQ + NAC	1.3357	0.44826		
Atazanavir/ritonavir + HCQ	1.6467	0.75201		
Atazanavir/ritonavir + HCQ + NAC	1.0733	0.28402		
AST				
Kaletra + HCQ	42.6923	15.93416	0.634	.597
Kaletra + HCQ + NAC	51.4167	45.73532		
Atazanavir/ritonavir + HCQ	46.1667	28.94457		
Atazanavir/ritonavir + HCQ + NAC	60.1333	42.96156		
ALT				
Kaletra + HCQ	28.3077	13.44981	1.114	.353
Kaletra + HCQ + NAC	46.2500	59.31599		
Atazanavir/ritonavir + HCQ	35.8333	24.87362		
Atazanavir/ritonavir + HCQ + NAC	52.3333	37.21303		
LDH				
Kaletra + HCQ	901.7143	222.52170	5.981	.003
Kaletra + HCQ + NAC	576.5000	170.14085		
Atazanavir/ritonavir + HCQ	694.6000	206.46622		
Atazanavir/ritonavir + HCQ + NAC	515.6250	188.30366		
CPK				
Kaletra + HCQ	294.8571	186.19562	0.771	.519
Kaletra + HCQ + NAC	272.4615	488.81380		
Atazanavir/ritonavir + HCQ	302.8571	293.94412		
Atazanavir/ritonavir + HCQ + NAC	639.1111	1006.70408		
CPK_M				
Kaletra + HCQ	28.3333	8.14453	0.198	.896
Kaletra + HCQ + NAC	20.6667	7.37111		
Atazanavir/ritonavir + HCQ	24.4286	9.50188		
Atazanavir/ritonavir + HCQ + NAC	24.4286	16.27736		
ALK_P				
Kaletra + HCQ	253.5000	243.59130	0.989	.407
Kaletra + HCQ + NAC	246.4000	108.58095		
Atazanavir/ritonavir + HCQ	153.7273	62.06463		
Atazanavir/ritonavir + HCQ + NAC	225.7143	137.50437		
bili_t				
Kaletra + HCQ	1.0875	0.87413	2.322	.093
Kaletra + HCQ + NAC	0.8750	0.32842		
Atazanavir/ritonavir + HCQ	0.7556	0.27889		
Atazanavir/ritonavir + HCQ + NAC	1.5154	0.97455		
bili_d				
Kaletra + HCQ	0.2875	0.13562	1.377	.266
Kaletra + HCQ + NAC	0.2875	0.13562		
Atazanavir/ritonavir + HCQ	0.2333	0.12247		
Atazanavir/ritonavir + HCQ + NAC	0.4538	0.42743		

Abbreviations: ALK_P, alkaline phosphatase; ALT, alanine aminotransferase; ANOVA, analysis of variance; AST, aspartate aminotransferase; bili_d, direct bilirubin; bili_t, total bilirubin; BUN, blood urea nitrogen; CPK, creatine phosphokinase; Cr, creatinine; CRP, C‐reactive protein; ESR, erythrocyte sedimentation rate; HCQ, hydroxychloroquine; HGB, hemoglobin; ICU, intensive care unit; INR, international normalized ratio; LDH, lactate dehydrogenase; NAC, *N*‐acetylcysteine; PLT, platelet; PT, prothrombin time; PTT, partial prothrombin time; RBC, red blood cell; WBC, white blood cell.

^a^ Statistics of one‐way ANOVA test.

^b^ All *p*‐values in this table are originated from ANOVA mean comparison.

**Table 2:** Clinical and paraclinical characteristics (quantitative variables) of the study groups at discharge time.

Variable	Mean	SD	*F* a	*p*‐Value^b^
WBC_dis				
Kaletra + HCQ	6.7167	3.38280	0.631	.600
Kaletra + HCQ + NAC	7.9231	3.07738		
Atazanavir/ritonavir + HCQ	7.9273	3.63045		
Atazanavir/ritonavir + HCQ + NAC	9.0200	3.52449		
diff_segment_dis				
Kaletra + HCQ	77.4500	9.16951	5.156	.007
Kaletra + HCQ + NAC	62.9714	9.53078		
Atazanavir/ritonavir + HCQ	78.4875	10.05861		
Atazanavir/ritonavir + HCQ + NAC	79.9600	5.87435		
diff_lymphocyte_dis				
Kaletra + HCQ	7.2667	8.98480	0.929	.433
Kaletra + HCQ + NAC	9.8800	13.09238		
Atazanavir/ritonavir + HCQ	7.3467	9.15883		
Atazanavir/ritonavir + HCQ + NAC	4.0000	6.25996		
seg_lymph_ratio_discharge				
Kaletra + HCQ	8.9700	7.69231	0.922	.445
Kaletra + HCQ + NAC	3.9786	2.45806		
Atazanavir/ritonavir + HCQ	12.4725	16.45986		
Atazanavir/ritonavir + HCQ + NAC	7.5540	3.34222		
RBC_dis				
Kaletra + HCQ	3.9717	0.66835	0.989	.409
Kaletra + HCQ + NAC	4.3777	0.76260		
Atazanavir/ritonavir + HCQ	4.3540	0.49552		
Atazanavir/ritonavir + HCQ + NAC	4.3740	0.70734		
HGB_dis				
Kaletra + HCQ	11.7500	1.85497	1.061	.378
Kaletra + HCQ + NAC	12.3154	1.95356		
Atazanavir/ritonavir + HCQ	13.1500	2.01674		
Atazanavir/ritonavir + HCQ + NAC	12.8800	1.90316		
PLT_dis				
Kaletra + HCQ	182.5833	92.96770	1.550	.219
Kaletra + HCQ + NAC	265.0000	89.59302		
Atazanavir/ritonavir + HCQ	256.0000	109.84231		
Atazanavir/ritonavir + HCQ + NAC	220.4000	137.21990		
ESR_dis				
Kaletra + HCQ	64.0000	0.00000	1.523	.317
Kaletra + HCQ + NAC	65.5000	0.70711		
Atazanavir/ritonavir + HCQ	30.2500	36.73668		
Atazanavir/ritonavir + HCQ + NAC	9.0000	–		
CRP_dis				
Kaletra + HCQ	40.0033	13.85929	9.102	.008
Kaletra + HCQ + NAC	36.0000	16.97056		
Atazanavir/ritonavir + HCQ	7.9967	3.46699		
Atazanavir/ritonavir + HCQ + NAC	5.9900	0.00000		
PT_dis				
Kaletra + HCQ	15.9000	2.73057	0.870	.478
Kaletra + HCQ + NAC	14.1000	1.23982		
Atazanavir/ritonavir + HCQ	14.8000	2.54558		
Atazanavir/ritonavir + HCQ + NAC	14.3333	2.59294		
INR_dis				
Kaletra + HCQ	1.2350	0.18738	1.985	.163
Kaletra + HCQ + NAC	1.0663	0.07671		
Atazanavir/ritonavir + HCQ	1.0000	–		
Atazanavir/ritonavir + HCQ + NAC	1.1000	0.17321		
PTT_dis				
Kaletra + HCQ	32.6667	5.57375	0.627	.609
Kaletra + HCQ + NAC	31.7500	4.13176		
Atazanavir/ritonavir + HCQ	32.3000	5.23259		
Atazanavir/ritonavir + HCQ + NAC	28.4000	1.75784		
BUN_dis				
Kaletra + HCQ	26.7500	16.55363	1.000	.403
Kaletra + HCQ + NAC	18.3000	7.48406		
Atazanavir/ritonavir + HCQ	20.0000	13.42318	–	–
Atazanavir/ritonavir + HCQ + NAC	21.5714	4.42934		
Cr_dis				
Kaletra + HCQ	1.1083	0.46409	1.690	.186
Kaletra + HCQ + NAC	1.2900	0.31429		
Atazanavir/ritonavir + HCQ	1.2417	0.49627		
Atazanavir/ritonavir + HCQ + NAC	0.8714	0.22887		
AST_dis				
Kaletra + HCQ	57.0000	44.49157	0.143	.868
Kaletra + HCQ + NAC	46.9000	34.40430		
Atazanavir/ritonavir + HCQ	49.0000	15.72683		
Atazanavir/ritonavir + HCQ + NAC	–	–		
ALT_dis				
Kaletra + HCQ	46.0000	42.13668	0.413	.668
Kaletra + HCQ + NAC	48.5000	44.10152		
Atazanavir/ritonavir + HCQ	68.7500	31.45764		
Atazanavir/ritonavir + HCQ + NAC	–	–		
LDH_dis				
Kaletra + HCQ	837.3333	497.61464	2.275	.115
Kaletra + HCQ + NAC	698.5000	235.20268		
Atazanavir/ritonavir + HCQ	500.5714	189.82435		
Atazanavir/ritonavir + HCQ + NAC	477.1667	78.03183		
CPK_dis				
Kaletra + HCQ	107.6667	81.98984	0.246	.862
Kaletra + HCQ + NAC	87.2500	52.42375		
Atazanavir/ritonavir + HCQ	85.2500	51.21442		
Atazanavir/ritonavir + HCQ + NAC	122.0000	35.35534		
CPK_mb_dis				
Kaletra + HCQ	55.0000	–		
Kaletra + HCQ + NAC	–	–		
Atazanavir/ritonavir + HCQ	10.0000	–		
Atazanavir/ritonavir + HCQ + NAC	–	–		
ALK_P_dis				
Kaletra + HCQ	56.9333	84.17878	7.650	.001
Kaletra + HCQ + NAC	137.2000	114.94048		
Atazanavir/ritonavir + HCQ	31.1333	82.19738		
Atazanavir/ritonavir + HCQ + NAC	0.0000	0.00000		
Bili_t_dis				
Kaletra + HCQ	1.3800	1.02567	2.648	.119
Kaletra + HCQ + NAC	0.8500	0.52820		
Atazanavir/ritonavir + HCQ	2.5500	1.62635		
Atazanavir/ritonavir + HCQ + NAC	–	–		
Bili_d_dis				
Kaletra + HCQ	0.4400	0.21909	0.455	.647
Kaletra + HCQ + NAC	0.3333	0.23381		
Atazanavir/ritonavir + HCQ	0.3000	0.00000		
Atazanavir/ritonavir + HCQ + NAC	–	–		

Abbreviations: ALK_P, alkaline phosphatase; ALT, alanine aminotransferase; ANOVA, analysis of variance; AST, aspartate aminotransferase; bili_d, direct bilirubin; bili_t, total bilirubin; BUN, blood urea nitrogen; CPK_M, creatine phosphokinase; Cr, creatinine; CRP, C‐reactive protein; _dis, at discharge; ESR, erythrocyte sedimentation rate; HCQ, hydroxychloroquine; HGB, hemoglobin; ICU, intensive care unit; INR, international normalized ratio; LDH, lactate dehydrogenase; NAC, *N*‐acetylcysteine; PLT, platelet; PT, prothrombin time; PTT, partial prothrombin time; RBC, red blood cell; WBC, white blood cell.

^a^ Statistics of one‐way ANOVA test.

^b^ All *p*‐values in this table are originated from ANOVA mean comparison.

Specifically, there were significant variations in CRP levels (*F* = 9.102, *p* = .008), alkaline phosphatase (ALP) levels (*F* = 7.650, *p* = .001), and differential segment (diff_segment) values (*F* = 5.156, *p* = .007) across the groups. The atazanavir/ritonavir + HCQ + NAC group exhibited the lowest CRP value, indicating a favorable outcome. In contrast, the Kaletra + HCQ + NAC group displayed the highest ALP value at discharge. Furthermore, the highest diff_segment value was observed in the atazanavir/ritonavir + HCQ + NAC group, suggesting distinctive patterns among the treatment groups.

Based on the *χ* ^2^ test, there were noteworthy differences in the utilization of intravenous immune globulin (IVIG) between the Kaletra + HCQ group (six patients) and the atazanavir/ritonavir + HCQ + NAC group (where no IVIG was administered), with this distinction proving statistically significant (*p* = .015). The initial random assignment of patients into four distinct groups led to discernible variations in terms of fatigue, anorexia, cardiovascular diseases, and hypertension, as outlined in Table 3.

**Table 3:** Clinical and paraclinical characteristics (qualitative variables) across the four study groups.

Variable	Group				*χ* ^2^ a	*p*‐Value^b^
	Kaletra + HCQ	Kaletra + HCQ + NAC	Atazanavir/ritonavir + HCQ	Atazanavir/ritonavir + HCQ + NAC		
Sex						
Female	9	5	8	9	2.870	.412
Male	6	10	7	6		
IVIG						
Negative	9	10	14	15	10.833	.015
Positive	6	5	1	0		
PCR						
Negative	11	11	9	10	3.487	.942
Positive	4	4	4	4		
Fever						
Negative	4	6	5	3	1.587	.662
Positive	11	9	10	12		
Cough						
Negative	4	4	4	1	2.369	.542
Positive	11	11	11	13		
Dyspnea						
Negative	5	3	2	1	3.498	.352
Positive	10	12	12	13		
Fatigue						
Negative	2	6	0	1	10.214	.013
Positive	11	9	15	13		
Anorexia						
Negative	1	10	2	3	17.434	.001
Positive	12	4	13	11		
Body pain						
Negative	5	5	3	3	1.200	.773
Positive	10	10	12	11		
Diarrhea						
Negative	9	15	13	13	6.584	.068
Positive	4	0	2	1		
Sore throat						
Negative	9	10	11	7	1.961	.624
Positive	4	5	4	7		
Sputum						
Negative	7	10	7	10	2.660	.485
Positive	7	5	8	4		
Chest discomfort						
Negative	6	13	9	9	5.287	.160
Positive	7	2	6	5		
Headache						
Negative	7	9	8	8	0.172	.982
Positive	6	6	7	6		
Vertigo						
Negative	10	14	8	11	6.638	.091
Positive	3	1	7	3		
Illusion						
Negative	8	10	12	13	4.236	.248
Positive	5	2	3	1		
Seizure						
Negative	13	12	15	14	2.716	.999
Positive	0	0	0	1		
LOC						
Negative	9	10	13	12	1.754	.690
Positive	4	2	2	2		
Smell loss						
Negative	11	7	12	12	4.930	.196
Positive	2	6	3	2		
Taste disorders						
Negative	13	8	12	12	6.659	.082
Positive	0	5	3	2		
Heart disease						
Negative	6	12	9	11	8.017	.044
Positive	9	3	6	2		
Lung disease						
Negative	11	13	12	14	2.400	.660
Positive	4	2	3	1		
Kidney disease						
Negative	10	14	11	13	5.576	.145
Positive	3	1	4	0		
Dialysis						
Negative	13	15	12	13	6.078	.166
Positive	0	0	2	0		
Immunodeficiency						
Negative	13	15	13	14	5.804	.237
Positive	0	0	2	0		
DM						
Negative	10	11	10	9	0.176	.999
Positive	4	4	5	4		
HTN						
Negative	5	13	9	11	8.547	.035
Positive	8	2	6	3		
Malignancy						
Negative	12	15	13	13	2.058	.681
Positive	1	0	2	1		
Dexamethasone						
Negative	15	10	6	3	21.991	.001
Positive	0	5	9	12		
Acetaminophen						
Negative	9	5	7	3	5.556	.152
Positive	6	10	8	12		
Azithromycin						
Negative	9	5	15	13	18.730	.001
Positive	6	10	0	2		
Ceftriaxone						
Negative	8	7	13	14	11.746	.007
Positive	7	8	2	1		
Heparin						
Negative	2	1	0	2	2.400	.740
Positive	13	14	15	13		
DiphenHCQamine						
Negative	8	11	11	9	1.978	.659
Positive	7	4	4	6		
Interferon‐β						
Negative	15	15	9	9	15.000	.002
Positive	0	0	6	6		
Levofloxacin						
Negative	7	5	13	5	11.467	.009
Positive	8	10	2	10		

Abbreviations: DM, diabetes mellitus; HCQ, hydroxychloroquine; HTN, hypertension; IVIG, intravenous immune globulin; LOC, level of consciousness; NAC, *N*‐acetylcysteine; PCR, polymerase chain reaction.

^a^ Statistics of *χ* ^2^ test.

^b^ All *p*‐values in this table are originated from crosstab (*χ* ^2^) frequency comparison.

In the realm of binary variables, the atazanavir/ritonavir + HCQ + NAC group demonstrated the highest incidence of fever (*n* = 12), cough (*n* = 13), and dyspnea (*n* = 13). Notably, fatigue was most frequently reported in both the atazanavir/ritonavir + HCQ + NAC group (*n* = 13) and the atazanavir/ritonavir + HCQ group (*n* = 13). Furthermore, the Kaletra + HCQ group accounted for 12 instances of body aches, while the Kaletra + HCQ group reported four cases of diarrhea. In the context of sore throat, the atazanavir/ritonavir + HCQ + NAC group documented seven occurrences, whereas chest discomfort was observed seven times in the Kaletra + HCQ group. Instances of headache were prevalent in the atazanavir/ritonavir + HCQ group (seven cases) and dizziness was notable in the Kaletra + HCQ + NAC group (14 cases). Interestingly, only one case of seizure was recorded in the atazanavir/ritonavir + HCQ + NAC group. Olfactory dysfunction was reported among six patients in the Kaletra + HCQ + NAC group (with the fewest cases noted in the Kaletra + HCQ + NAC and atazanavir/ritonavir + HCQ + NAC groups), while five instances of taste disorders were identified in the Kaletra + HCQ + NAC group.

The utilization of dexamethasone, acetaminophen, azithromycin, ceftriaxone, interferon‐β, and levofloxacin differed across the four groups. For instance, within the atazanavir/ritonavir + HCQ and atazanavir/ritonavir + HCQ + NAC groups, six patients each were administered interferon‐β (*p* = .002), which contributed to the absence of mortality in the atazanavir/ritonavir + HCQ + NAC group. Although two patients in the Kaletra + HCQ group, one patient in the atazanavir/ritonavir + HCQ group, and one patient in the Kaletra + HCQ + NAC group did not survive, this distinction was not statistically significant (*χ* ^2^ = 2.134, *p* = .896). Notably, all patients within the atazanavir/ritonavir + HCQ + NAC group were discharged in favorable health conditions (refer to Table 4).

**Table 4:** Comparison of the final condition between the four study groups receiving four types of medicine.

Group	Final condition		*χ* ^2^ a	*p*‐Value^b^
	Died *n* (%)	Discharged *n* (%)		
Kaletra + HCQ	2 (50%)	13 (23.2%)	2.134	.896
Kaletra + HCQ + NAC	1 (25%)	14 (25%)		
Atazanavir/ritonavir + HCQ	1 (25%)	14 (25%)		
Atazanavir/ritonavir + HCQ + NAC	–	15 (26.8%)		
Total	4 (100%)	56 (100%)		

Abbreviations: HCQ, hydroxychloroquine; NAC, *N*‐acetylcysteine.

^a^ Statistics of *χ* ^2^ test.

^b^ All *p*‐values in this table are originated from crosstab (*χ* ^2^) frequency comparison.

**Figure 1:**
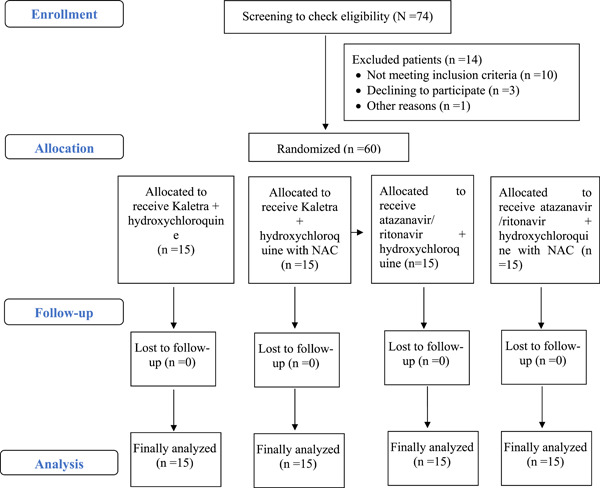
The flow diagram shows the flow of patients through the trial.

Applying a linear regression model to forecast ICU stay duration, variables including the usage of IVIG, elevation of creatine phosphokinase (CPK), and decrease in ESR were associated with prolonged ICU stays (see Table 5). No significant discrepancies in terms of mortality were observed among the various medications employed (see Table 6 and Figure 1).

**Table 5:** Linear regression to predict ICU days as a dependent variable.

Variable	Unstandardized coefficients		Standardized coefficients	*p*‐Value^a^
	*β*	Standard error	*β* b	
Gender	2.245	1.762	.327	.212
Age	.036	0.045	.454	.438
O_2__sat_before	−.045	0.070	−.805	.528
NAC	−.440	1.658	−.065	.792
Atazanavir/ritonavir	21.483	17.421	3.186	.227
Lopinavir/ritonavir	24.658	18.230	3.657	.186
IVIG	5.910	2.864	.554	.048
PCR	−.969	1.502	−.115	.524
WBC	−.025	0.069	−.071	.719
diff_segment	−.012	0.051	−.194	.812
diff_lymphocyte	−.223	0.171	−.973	.204
seg_lymph_ratio	−.842	0.447	−1.214	.070
RBC	−3.689	2.004	−3.529	.076
HGB	1.144	0.750	3.155	.138
PLT	.007	0.009	.303	.472
ESR	−.096	0.042	−1.104	.031
CRP	−.012	0.057	−.093	.837
PT	−.600	1.225	−1.822	.628
INR	.361	9.449	.089	.970
PTT	−.014	0.119	−.107	.906
BUN	−.018	0.088	−.092	.838
Cr	−1.516	1.733	−.499	.388
AST	−.013	0.056	−.160	.821
ALT	.011	0.056	.122	.847
LDH	.003	0.005	.465	.542
CPK	.006	0.003	.713	.034
CPK_M	−.090	0.162	−.479	.582
ALK_P	.013	0.008	.688	.108
Bilirubin total	.595	2.600	.158	.821
Bilirubin direct	1.641	7.130	.137	.819

Abbreviations: ALK_P, alkaline phosphatase; ALT, alanine aminotransferase; AST, aspartate aminotransferase; BUN, blood urea nitrogen; CPK_M, creatine phosphokinase; Cr, creatinine; CRP, C‐reactive protein; ESR, erythrocyte sedimentation rate; HGB, hemoglobin; ICU, intensive care unit; INR, international normalization ratio; IVIG, intravenous immune globulin; LDH, lactate dehydrogenase; PCR, polymerase chain reaction; PLT, platelet; PT, prothrombin time; PTT, partial prothrombin time; RBC, red blood cell; _sat, saturation; WBC, white blood cell.

^a^ All *p*‐values in this table are originated from linear regression for prediction.

^b^ Standardized coefficients of linear regression.

**Table 6:** Comparison of the study patients' final condition according to the drugs administered.

Variable	Final condition		*χ* ^2^ a	*p*‐Value^b^
	Died	Discharged		
Dexamethasone				
Negative	3	31	0.587	.626
Positive	1	25		
Acetaminophen				
Negative	2	22	0.179	.999
Positive	2	34		
Azithromycin				
Negative	4	38	1.837	.306
Positive	0	18		
Ceftriaxone				
Negative	2	40	0.816	.576
Positive	2	16		
Heparin				
Negative	0	5	0.390	.999
Positive	4	51		
Diphenhidramine				
Negative	2	37	0.424	.606
Positive	2	19		
Interferon‐β				
Negative	4	44	1.071	.574
Positive	0	12		
Levofloxacin				
Negative	1	29	1.071	.612
Positive	3	27		

^a^ Statistics of *χ* ^2^ test.

^b^ All *p*‐values in this table are originated from crosstab (*χ* ^2^) frequency comparison.

**Figure 2:**
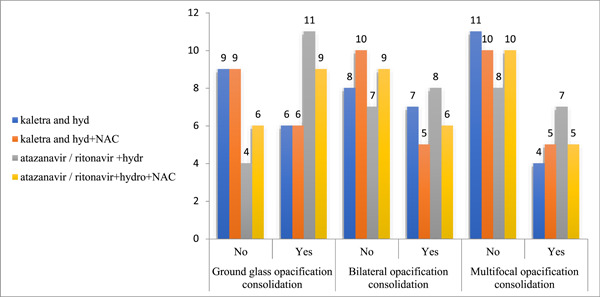
The image illustrates a comparison of opacification consolidation between the study groups according to the drugs administered.

Regarding the enhancement of oxygen saturation (O_2_ saturation) brought about by the four different treatment protocols, the findings indicated that both the atazanavir/ritonavir + HCQ + NAC group (*p* = .001) and the atazanavir/ritonavir + HCQ group (*p* = .008) demonstrated notable improvements. These groups exhibited a substantial increase in O_2_ saturation levels posttreatment, in comparison to the initial O_2_ saturation levels before intervention (as shown in Table 7). However, no statistically significant variations were noted in this aspect among the four groups (*p* > .05), as detailed in Table 8 and illustrated in Figure 2.

**Table 7:** Comparison of O_2_ saturation levels between the four study groups receiving four types of medicine.

Variable	Mean	SD	*t* a	*p*‐Value^b^
Lopinavir/ritonavir + HCQ + NAC				
O_2__sat_ before	89.73	4.250	0.655	.523
O_2__ sat_ after	88.6667	7.73366		
Lopinavir/ritonavir + HCQ				
O_2__sat_before	89.80	6.689	1.817	.091
O_2__ sat_ after	79.7333	21.45582		
Atazanavir/ritonavir + HCQ				
O_2__sat_before	70.87	21.722	−2.898	.008
O_2__ sat_ after	85.3478	9.62765		
Atazanavir/ritonavir + HCQ + NAC				
O_2__sat_before	74.56	13.395	−4.138	.001
O_2__ sat_ after	85.9444	6.02419		

Abbreviations: HCQ, hydroxychloroquine; NAC, *N*‐acetylcysteine; _sat, saturation.

^a^ Statistics of independent *t*‐test.

^b^ All *p*‐values in this table are originated from independent t‐test for two groups mean comparison.

**Table 8:** Comparison of the opacification consolidation between the study groups according to the drugs administered.

Variable	Kaletra + HCQ	Kaletra + HCQ + NAC	Atazanavir/ritonavir + HCQ	Atazanavir/ritonavir + HCQ + NAC	*χ* ^2^ a	*p*‐Value^b^
Ground glass opacification consolidation						
No	9	9	4	6	4.821	.225
Yes	6	6	11	9		
Bilateral opacification consolidation						
No	8	10	7	9	1.357	.716
Yes	7	5	8	6		
Multifocal opacification zz						
No	11	10	8	10	1.392	.778
Yes	4	5	7	5		

Abbreviations: HCQ, hydroxychloroquine; NAC, *N*‐acetylcysteine.

^a^ Statistics of *χ* ^2^ test.

^b^ All *p*‐values in this table are originated from crosstab (*χ* ^2^) frequency comparison.

## 4 DISCUSSION

There is accumulating evidence pointing toward the therapeutic potential of NAC in addressing COVID‐19 and its associated repercussions. For instance, NAC's impact on oxidative stress regulation, immune modulation, and apoptosis management, combined with its unique attributes such as enhancing oxygenation and circulation, can significantly contribute to improved respiratory outcomes and the prevention of end‐organ failure. Furthermore, well‐designed studies have highlighted NAC's roles as an antioxidant and immunomodulator in combatting viruses that target the respiratory system, such as influenza strains A and B, and the respiratory syncytial virus. This extends to addressing acute injuries like ARDS.

Notably, NAC's potential extends beyond its supportive role in ICU patients, those with sepsis, and individuals with nonpulmonary end‐organ complications. It may also offer positive contributions to patients with underlying health conditions. Moreover, NAC could serve as a promising supplementary therapeutic option for COVID‐19, taking into account patient conditions, indications, and contraindications.^5^, ^32^, ^33^, ^34^ Oral NAC could potentially serve as a preventive or treatment option for disease‐related outcomes in stable patients who are not experiencing sepsis and are not reliant on intubation. Intravenous (IV) administration of NAC has shown promise in moderate‐to‐severe cases of COVID‐19, particularly among individuals admitted to the ICU with complications like end‐organ failure. Numerous reports have highlighted the effectiveness of NAC in managing cytokine storms, alleviating dyspnea, and addressing ARDS associated with COVID‐19.^5^ The utilization of NAC through different administration routes is contingent upon the specific context. At present, drawing from studies with robust evidence, it can be deduced that NAC's efficacy is particularly notable in stable patients when administered at the standard dose. Its most significant impact might lie in its preventive capacity—meaning its potential to improve the disease trajectory for noninfected individuals or those already infected. This multifaceted drug's primary role could thus be its ability to preemptively intervene.^35^ To our current understanding, this study stands out as one of the most well‐designed RCTs conducted thus far to assess the effectiveness and safety of NAC in hospitalized patients afflicted with COVID‐19 infection. In brief, the results of this RCT revealed that the average duration of hospitalization (not confined to the ICU) was the shortest among individuals in the atazanavir/ritonavir + HCQ group (6.73 days) and the longest in those in the Kaletra + HCQ + NAC group (11.87 days). However, the discrepancies in hospitalization duration among the four treatment groups did not attain statistical significance (*p* = .082). Correspondingly, the mean duration of ICU stay was notably briefer for patients in the atazanavir/ritonavir + HCQ + NAC group (1.8 days) compared to the Kaletra + HCQ group (3.4 days), although these differences did not achieve statistical significance either (*p* = .172). Despite the absence of statistical significance, these outcomes suggest that patients administered with NAC exhibited more total hospitalization days and fewer ICU hospitalization days, implying a greater likelihood of maintaining a stable overall condition.

Upon hospitalization and before the initiation of the primary treatment, analysis of CT scans and severity scores indicated that ground glass opacification consolidation was most prevalent in the atazanavir/ritonavir + HCQ group (*n* = 11), and least common in the Kaletra + HCQ + NAC and Kaletra + HCQ groups (*n* = 6). Similarly, bilateral opacification consolidation was most frequent in the atazanavir/ritonavir + HCQ group (*n* = 8) and least frequent in the Kaletra + HCQ + NAC group (*n* = 5). The highest frequency of multifocal opacification consolidation was observed in the atazanavir/ritonavir + HCQ group (*n* = 7), and the lowest in the Kaletra + HCQ group (*n* = 4). Notably, these variations did not show any significant differences among the groups, indicating a relatively uniform distribution of lung involvement severity across the treatment groups within this RCT.

Upon hospitalization and preceding the initiation of the primary treatment, there were statistically significant differences among the four study groups in terms of seg_lymph_ratio, CRP, partial thromboplastin time, and LDH. However, at the conclusion of the study, only CRP exhibited sustained statistically significant disparities between the groups. Notably, the NAC group demonstrated a notable reduction in CRP levels by the study's end. Noteworthy was the observation that at discharge, the atazanavir/ritonavir + HCQ + NAC group displayed the lowest CRP value (5.99), while the Kaletra + HCQ group recorded the highest value (40.00). This disparity in CRP levels held statistical significance (*p* = .008). As for ESR at discharge, the atazanavir/ritonavir + HCQ + NAC group showed the least value (9.00), whereas the Kaletra + HCQ + NAC group exhibited the highest value (65.50). However, this discrepancy did not manifest as statistically significant, suggesting that CRP changes serve as a more sensitive measure of treatment response and inflammation reduction compared to ESR.

Outside of CRP, the statistically significant comparisons between laboratory findings upon hospitalization and at discharge are as follows:

For diff_segment at discharge, the Kaletra + HCQ + NAC group reported the lowest value (62.97%), while the atazanavir/ritonavir + HCQ + NAC group exhibited the highest value (79.96%).

In terms of ALP at discharge, the highest and lowest values were observed in the Kaletra + HCQ + NAC group and the atazanavir/ritonavir + HCQ + NAC group, respectively. This discrepancy held statistical significance (*p* = .001). This suggests that the atazanavir/ritonavir regimen exhibited a more pronounced effect on diff_segment and ALP, indicating a more substantial improvement trend compared to the Kaletra (lopinavir/ritonavir) regimen.

In the context of mortality, the absence of any deaths in the atazanavir/ritonavir + HCQ + NAC group hints at the potential for reduced mortality when these drugs are combined.

With regard to O_2_ saturation, the highest levels were noted in the atazanavir/ritonavir + HCQ group and the atazanavir/ritonavir + HCQ + NAC group at the study's conclusion. Furthermore, a significant increase in O_2_ saturation was observed post‐intervention in groups that received NAC (*p* < .05), marking a pivotal finding in this study.

The majority of COVID‐19 cases are characterized by a redox imbalance in alveolar epithelial cells, triggering apoptosis, heightened inflammation, and consequent impairment of gas exchange. Numerous studies have discussed the potential beneficial effects of NAC as a versatile drug in managing COVID‐19 and its associated complications.^5^ Furthermore, several primary investigations encompassing case reports, case series, and clinical trials have directed their attention towards the utilization of NAC in the therapeutic approach and care of patients afflicted with COVID‐19, along with its accompanying complications. These complications encompass end‐organ failure, particularly instances of acute liver failure stemming from factors such as remdesivir‐induced liver dysfunction, heightened liver enzyme levels, and occurrences of intrahepatic hemorrhage. Additionally, NAC's potential has been explored in the management of ARDS and the mitigation of seizure occurrences.^36^, ^37^

A RCT was conducted in Brazil with the aim of assessing the effectiveness and safety of IV NAC in severe cases of COVID‐19, defined by oxyhemoglobin saturation falling below 94% or a respiratory rate surpassing 24 breaths/min. The results of this trial indicated that IV NAC did not yield a significant reduction in the requirement for mechanical ventilation when compared to the control group. Specifically, 20.6% of individuals in the NAC group necessitated mechanical ventilation, as opposed to 23.9% in the control group.

Furthermore, the trial outcomes revealed that various parameters, including the duration of mechanical ventilation, the frequency of ICU admission, the length of ICU stays, and the rate of mortality, did not demonstrate statistically significant differences between the group receiving NAC intervention and the control group. These results collectively suggest that the administration of high doses of NAC did not influence the progression of severe COVID‐19.^35^ The current study exclusively enrolled stable COVID‐19 patients categorized as moderate‐to‐severe, who were not admitted to the ICU (*N* = 60). Among them, only 11 patients required ICU admission during the course of their illness, regardless of the treatment regimen they were on. It is worth noting that the patients' severity scores and the mode of NAC administration (IV vs. oral) differed between our present RCT and the one conducted in Brazil. Despite these differences, the interpretation of findings from both trials suggests that NAC might exhibit greater effectiveness as a preventive or supplementary therapy in stable, nonsevere cases of COVID‐19. It particularly seems to play a positive role in improving oxygen saturation levels and hastening the reduction of CRP levels and inflammation.

Beyond NAC's therapeutic applications, which include its use as an adjunct therapy, the potential prophylactic benefits of this versatile drug in the context of COVID‐19 infection are also of notable significance^38^, ^39^, ^40^ and its related complications^41^ have been discussed in many reviews^40^, ^42^, ^43^, ^44^, ^45^, ^46^, ^47^ and original studies^48^, ^49^ all of which have focused mainly on the drug as an anti‐inflammatory and antiapoptotic agent.

In a meticulously designed study examining the use of NAC for treating COVID‐19 patients, high doses of the drug did not result in improved outcomes for cases classified as severe and requiring admission to the ICU.^35^, ^50^ An additional clinical trial showcased that the amalgamation of methylene blue, vitamin C, and NAC led to an elevated survival rate among patients with COVID‐19.^16^ Findings from a series of cases suggested that both oral and IV administration of glutathione, along with glutathione precursors like *N*‐acetyl‐cysteine and α‐lipoic acid, could potentially offer a new therapeutic avenue for inhibiting nuclear factor kappa B (NF‐κB), managing cytokine storms, and addressing ARDS in individuals diagnosed with COVID‐19 pneumonia.^51^ The outcomes of a case series propose that a fresh therapeutic approach might be viable through the oral and IV application of glutathione, in addition to its precursors like *N*‐acetyl‐cysteine and α‐lipoic acid. This approach shows promise in potentially curtailing NF‐κB activity, regulating cytokine storms, and addressing the onset of ARDS among individuals diagnosed with COVID‐19 pneumonia.^17^ The findings of a case series suggest the potential for a novel therapeutic strategy involving the oral and IV administration of glutathione, along with its precursors like *N*‐acetyl‐cysteine and α‐lipoic acid. This approach holds promise in potentially attenuating NF‐κB activity, modulating cytokine storms, and mitigating the onset of ARDS in individuals diagnosed with COVID‐19 pneumonia.^52^, ^53^, ^54^, ^55^, ^56^, ^57^, ^58^, ^59^, ^60^, ^61^, ^62^, ^63^ They have also undertaken exhaustive and methodical analyses, both in the form of systematic reviews and original articles. Their initial review study delved into potential drugs that might exert a positive influence on the progression and outcomes of COVID‐19, including NAC.^5^ Following this, their efforts have been directed toward conducting RCTs aimed at assessing the effectiveness and safety of versatile drugs like NAC. Building upon the conclusions of the present RCT, we postulate that the utilization of oral or IV NAC, as appropriate, could potentially enhance oxygen saturation, mitigate inflammation by reducing CRP levels, and contribute to a reduction in mortality rates.

## 5 CONCLUSIONS

In the context of the current absence of a definitive gold‐standard therapy for COVID‐19 and its associated complications, the potential of multipotential drugs endowed with anti‐inflammatory, antioxidant, and antiapoptotic attributes emerges as a promising avenue when appropriately administered. Our study stands as one of the most meticulously designed RCTs to date, with the purpose of assessing the safety and efficacy of NAC in the treatment of COVID‐19 patients.

The pivotal outcomes gleaned from this RCT highlight that, upon study conclusion, the NAC group exhibited a noteworthy additional reduction in CRP levels (*p* = .008). Notably, within the atazanavir/ritonavir + HCQ + NAC group, there were no reported instances of mortality, suggesting a potential for the combined administration of these medications to mitigate mortality risk. Furthermore, both the atazanavir/ritonavir + HCQ group and the atazanavir/ritonavir + HCQ + NAC group displayed the highest levels of oxygen saturation at the study's termination, alongside a substantial elevation in oxygen saturation subsequent to the initiation of the intervention, including NAC (*p* < .05).

Considering the insights derived from this RCT, we can affirm that oral NAC, when appropriately indicated, holds the potential to enhance oxygen saturation levels, temper the trajectory of inflammation (via CRP reduction), and contribute to a decrease in mortality risk among hospitalized COVID‐19 patients. Notably, NAC may exhibit heightened efficacy as a prophylactic or adjunctive therapy in cases of stable nonsevere COVID‐19, with a particularly positive role in augmenting oxygen saturation levels and expediting the reduction of CRP and associated inflammation.

## 6 LIMITATIONS

In this randomized clinical trial evaluating the efficacy and safety of oral NAC in COVID‐19 patients undergoing the routine antiviral and HCQ protocol, we recognize several limitations that warrant consideration when interpreting our findings.

1. Low sample size: A prominent limitation of our study is the relatively modest sample size within each treatment group. The restricted number of participants compromises the statistical power of our analysis and may hinder the detection of subtle treatment effects. As such, caution must be exercised when generalizing our results to broader patient populations.
2. Heterogeneous baseline characteristics: The differences in background characteristics among the treatment groups introduce variability that might confound our results. We acknowledge that variations in demographics, medical history, and comorbidities can influence treatment responses, thereby limiting the direct comparability of outcomes. Although we have reported these disparities transparently, the challenge of unequal baseline data remains a weakness in our study design.
3. Limited serial laboratory parameter evaluations: The importance of serially evaluating laboratory parameters, including TNF and IL‐6, as integral components of response‐to‐treatment criteria, is acknowledged. Unfortunately, financial constraints hindered the incorporation of these tests, which would have provided comprehensive insights into the autoinflammatory response to treatments. While acknowledging this limitation, we have focused on assessing feasible parameters within the confines of our available resources.

Despite these limitations, our study offers valuable insights into the potential benefits and challenges of incorporating NAC into the routine antiviral and HCQ protocol for COVID‐19 patients. We emphasize our commitment to transparency by candidly discussing these limitations, enabling readers to interpret our results within the context of these constraints. While our findings contribute to the current understanding, we acknowledge the need for future investigations with larger and more homogenous samples to establish more robust conclusions regarding the role of NAC in COVID‐19 treatment protocols.

## AUTHOR CONTRIBUTIONS

Azadeh Goodarzi and Najmolsadat Atefi conceived and planned the intervention. Taghi Riahi, Niloofar Khodabandehloo, Mahshid Talebi Taher and Niloufar Najar Nobari carried out the intervention. Zeinab Mahdi and Amir Baghestani gathering the datas. Amir Baghestani, Zeinab Mahdi, Rohollah Valizadeh, and Farnoosh Seirafianpour contributed to the interpretation of the results. Rohollah Valizadeh analyzed the data. Farnoosh Seirafianpour took the lead in writing the manuscript. All authors contributed to the preparation of data and the finalization of this article. All the figures have been produced by the authors of this article and are personal data.

## CONFLICT OF INTEREST STATEMENT

The authors declare no conflict of interest.
